# Supplementary material for: FoxO3 Modulates Circadian Rhythms in Neural Stem Cells
Source: Int J Mol Sci. 2023 Sep 4;24(17):13662. doi: 10.3390/ijms241713662 (PMC10563086; doi:10.3390/ijms241713662)
Supplement: Supplementary file 1 [file ijms-24-13662-s001.zip › ijms-2554277-supplementary.pdf]

Supplementary Material

FoxO3 modulates circadian rhythms in neural stem cells

Swip Draijer[1], Raissa Timmerman[1], Jesse Pannekeet[1], Alexandra van Harten[1], Elham Farshadi[2], Julius Kemmer[2], Demy van Gilst[2], Inês Chaves[2#*] and Marco F.M. Hoekman[1#]

[1] Swammerdam Institute of Life Sciences, University of Amsterdam, Amsterdam, the Netherlands
[2] Department of Molecular Genetics, Erasmus University Medical Center, Rotterdam, the Netherlands

# shared last authors
* Correspondence: Inês Chaves (i.chaves@erasmusmc.nl)

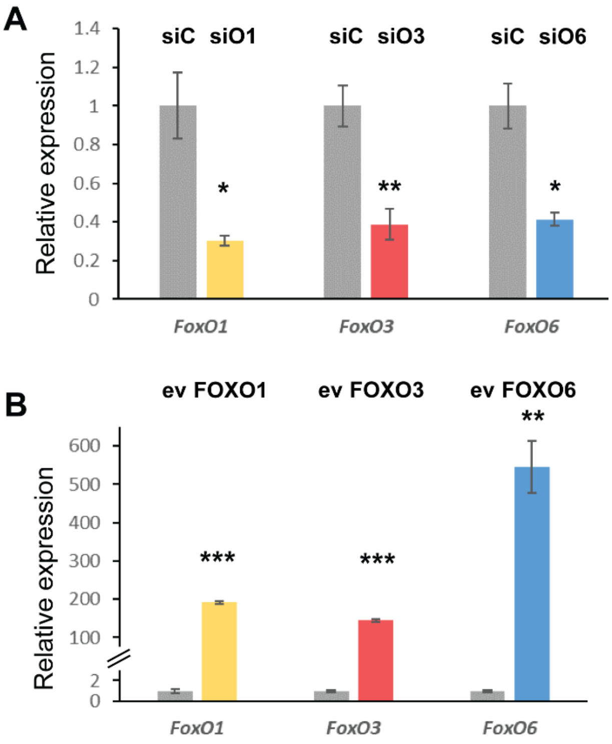

Figure S1. Quantification of *FoxO* expression levels in the experiments described in Figure 1. Relative expression of *FoxO1*, *FoxO3* and *FoxO6* upon siRNA knockdown or overexpression of either *FoxO1* (yellow), *FoxO3* (red) or *FoxO6* (blue). Control siC and ev are shown in grey. Relative expression measured by RT-qPCR. *p<0.05, **; p<0.01; ***, p<0.001.

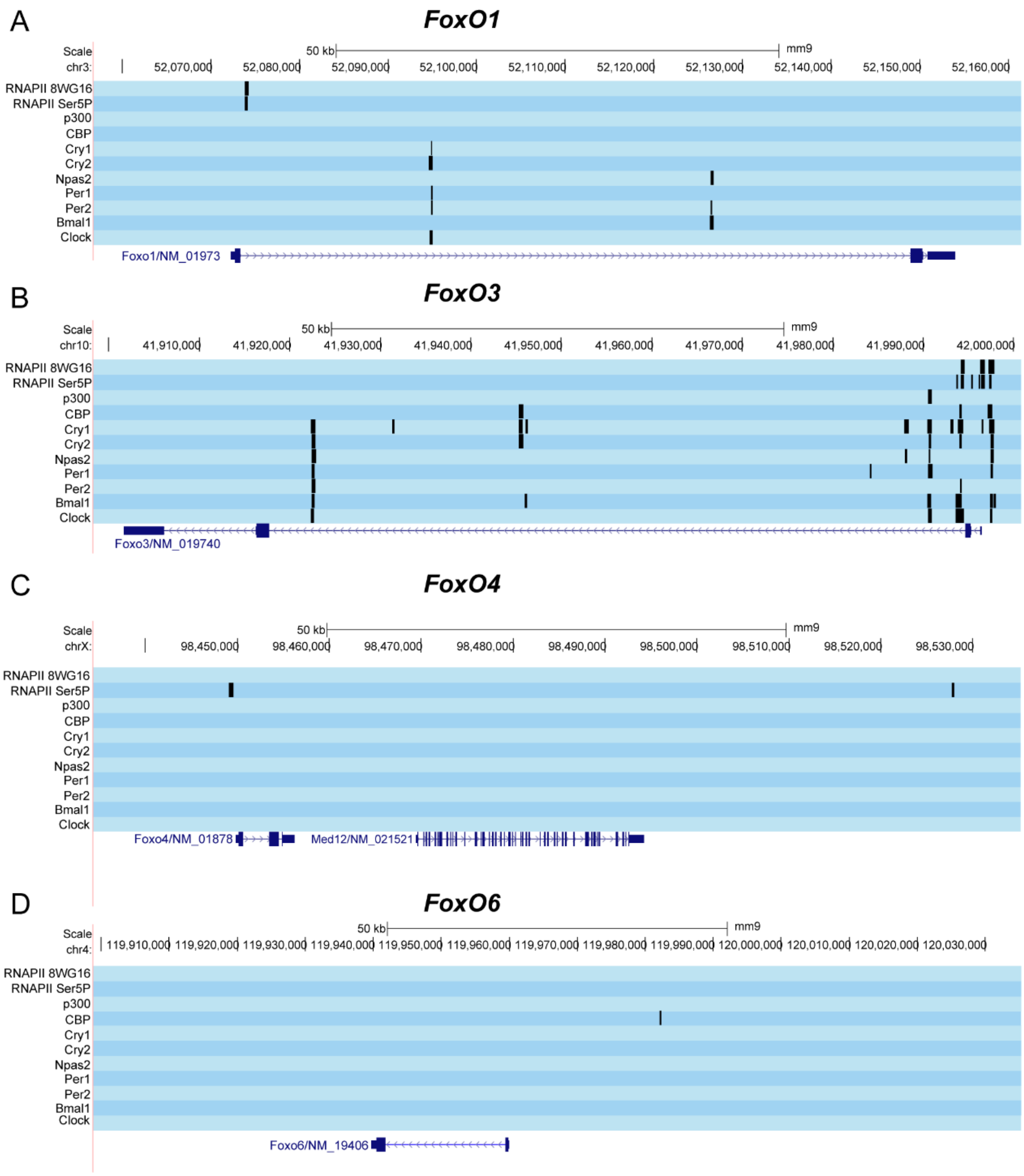

Figure S2. Differential binding of circadian clock components and transcriptional regulators to FoxO transcription factors. University of California Santa Cruz (UCSC) genome browser track view of the *FoxO1* **(A)**, *FoxO3* **(B)**, *FoxO4* **(C)** and *FoxO6* **(D)** genes showing ChIP-seq binding peaks for circadian clock components Clock, Bmal1, Per1, Per2, Npas2, Cry1 and Cry2 as well as transcriptional regulators CBP, p300 and recruitment and initiation of RNA polymerase II (8WG16 and Ser5P respectively). Data from [S1].

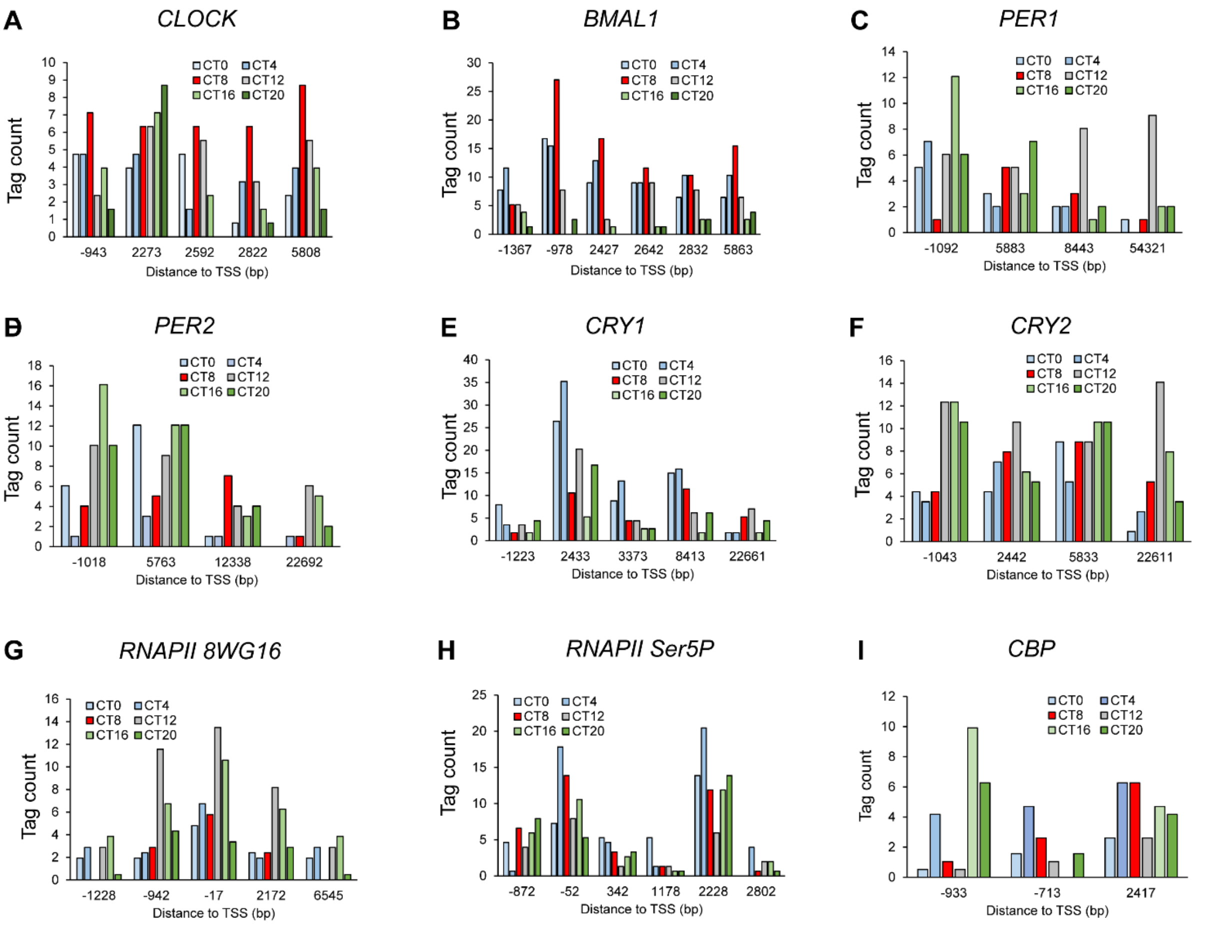

Figure S3. Circadian occupancy of FoxO3 by circadian clock components and transcriptional regulators. Occupancy on multiple regions within the FoxO3 gene during 24 hours: (A) CLOCK; (B) BMAL1; (C) PER1; (D) PER2; (E) CRY1; (F) CRY2; (G) RNA polymerase II 8WG16 (recruitment); (H) RNA polymerase II Ser5P (initiation); (I) CBP. Data is from [S1].

Supplementary References

S1. Koike, N., Yoo, S. H., Huang, H. C., Kumar, V., Lee, C. et al. (2012). Transcriptional architecture and chromatin landscape of the core circadian clock in mammals. Science, 338, 349–354.
